# Supplementary material for: A Comparison of the Meat Quality, Nutritional Composition, Carcass Traits, and Fiber Characteristics of Different Muscular Tissues between Aged Indigenous Chickens and Commercial Laying Hens
Source: Foods. 2023 Oct 7;12(19):3680. doi: 10.3390/foods12193680 (PMC10573064; doi:10.3390/foods12193680)
Supplement: Supplementary file 1 [file foods-12-03680-s001.zip › foods-2597494-supplementary.pdf]

Table S1. Dietary composition and nutritional content of HLG and GYG.

Ingredient (%)	Rearing stage (%)	Laying stage (%)
Corn	63.00	59.80
Soybean meal (sol.)	11.50	12.80
Maize gluten meal	3.00	5.00
Wheat bran	7.00	4.40
Rapeseed meal (sol.)	8.00	7.00
Soybean oil	2.80	3.30
Dicalcium phosphate	1.20	1.00
Limestone	1.00	4.20
Premixes [a]	2.50	2.50
Nutrition content		
ME（MJ/Kg）	12.15	12.14
CP（%）	16.22	17.01
Calcium（%）	0.85	2.00
Available P（%）	0.4	0.34
Lys（%）	0.64	0.66
Met（%）	0.29	0.31

[a] Premix included the following per kg of feed: Vitamin A ≥ 390 KIU, Vitamin D3 ≥ 150 KIU, Vitamin E ≥ 1240, Vitamin B ≥ 185, Vitamin B2 ≥ 260, Vitamin B6 ≥ 160, Vitamin B12 ≥ 1.2, Vitamin K3 ≥ 100, D-biotin ≥ 12, D-pantothenic acid ≥ 470, Folic acid ≥ 57, niacin ≥ 1660, Hydrogenated choline ≥ 15,000, methionine ≥ 43,000, Fe ≥ 2000, Cu ≥ 380, Mn ≥ 3900, Zn ≥ 2800, I ≥ 25, Se ≥ 10.
